# Optimizing functional near-infrared spectroscopy (fNIRS) channels for schizophrenic identification during a verbal fluency task using metaheuristic algorithms

**DOI:** 10.3389/fpsyt.2022.939411

**Published:** 2022-07-18

**Authors:** Dong Xia, Wenxiang Quan, Tongning Wu

**Affiliations:** ^1^China Academy of Information and Communications Technology, Beijing, China; ^2^Peking University Sixth Hospital, Peking University Institute of Mental Health, NHC Key Laboratory of Mental Health (Peking University), National Clinical Research Center for Mental Disorders (Peking University Sixth Hospital), Beijing, China

**Keywords:** schizophrenia, functional near-infrared spectroscopy (fNIRS), verbal fluency task (VFT), metaheuristic algorithms, support vector machine (SVM)

## Abstract

**Objective:**

We aimed to reduce the complexity of the 52-channel functional near-infrared spectroscopy (fNIRS) system to facilitate its usage in discriminating schizophrenia during a verbal fluency task (VFT).

**Methods:**

Oxygenated hemoglobin signals obtained using 52-channel fNIRS from 100 patients with schizophrenia and 100 healthy controls during a VFT were collected and processed. Three features frequently used in the analysis of fNIRS signals, namely time average, functional connectivity, and wavelet, were extracted and optimized using various metaheuristic operators, i.e., genetic algorithm (GA), particle swarm optimization (PSO), and their parallel and serial hybrid algorithms. Support vector machine (SVM) was used as the classifier, and the performance was evaluated by ten-fold cross-validation.

**Results:**

GA and GA-dominant algorithms achieved higher accuracy compared to PSO and PSO-dominant algorithms. An optimal accuracy of 87.00% using 16 channels was obtained by GA and wavelet analysis. A parallel hybrid algorithm (the best 50% individuals assigned to GA) achieved an accuracy of 86.50% with 8 channels on the time-domain feature, comparable to the reported accuracy obtained using 52 channels.

**Conclusion:**

The fNIRS system can be greatly simplified while retaining accuracy comparable to that of the 52-channel system, thus promoting its applications in the diagnosis of schizophrenia in low-resource environments. Evolutionary algorithm-dominant optimization of time-domain features is promising in this regard.

## Introduction

Schizophrenia is a chronic, frequently disabling mental disorder (1). Patients with schizophrenia (SZs) can display both visual and phonological impairments (2), which are vital for the successful development/attainment of literacy skills, resulting in deficits in rapid naming and phonological awareness (3, 4). Schizophrenia results in mixed deficits in morphological, orthographical, and phonological processing skills (5–8) depending on their native language. Therefore, the verbal fluency task (VFT), a language-related neuropsychological task, has been employed for discriminating schizophrenia (9). During the VFT, the participant was asked to make as many phrases as possible, starting with the character or letter appearing on the screen. The phrases could be made based on the semantic or phonological approach. Neuroimaging techniques were used to detect the hemodynamic changes or neuronal firing during the task. Among them, functional near-infrared spectroscopy (fNIRS) can noninvasively measure hemodynamic signals from the cortex (10), and derive underlying neuronal networks and functional connectivity (11). Although the spatial resolution of fNIRS is relatively low (most current fNIRS system has 52 channels), it has advantages on portability and cost, which make fNIRS popular in schizophrenic studies, especially in developing countries with poor medical resources.

Signals from multiple channels enhance the classification capability but at the cost of increased complexity, making it difficult to move and preventing its extensive usage in clinical situations of low-resource environments. Therefore, researchers have attempted channel simplification. Our group obtained a classification accuracy of 85.83% [120 SZs and 120 healthy controls (HCs)] with 11 components by using principal component analysis on time-varying features (12); however, each component was a linear combination of signals from dozens of channels. In this study, the performance of four machine-learning classifiers, namely linear discriminant analysis, k-nearest neighbors, Gaussian process classifier, and support vector machine (SVM), were assessed; the results revealed that SVM performed the best. By using the same dataset, Ji et al. (13) and Yang et al. (14) conducted seed-based analysis on functional connectivity (FC) for classification and achieved an accuracy of 89.67% with 26 channels. Einalou et al. (15) and Dadgostar et al. (16) achieved an accuracy exceeding 84.00% with eight channels after selecting from a 16-channel fNIRS by using genetic algorithm (GA) and wavelet analysis. However, these studies involved only 16 participants. Chen et al. (17) achieved an accuracy of 89.5% with 39 channels by using the general linear model on time-domain features. These results indicate that redundancy exists in multichannel signals, and this redundancy can be reduced by applying appropriate optimization methods.

Metaheuristic optimization is based on functional evaluation and relies less on the properties of objective functions and constraints. This method does not take advantage of the specificity of the targeted problem and is thus widely used in physiological signal processing. These optimization processes generally use a set of solutions inspired by a certain natural analogy or philosophy. As mentioned earlier, a preliminary trial with GA, an evolutionary algorithm, was carried out for 16 participants (15, 16). In effect, the searching process employed by GA is, to some extent, omnidirectional because the crossover and mutation are randomly initiated. This may degrade the exploration ability, although a faster convergence can be achieved. In nature, animals' foraging behavior is not only under genetic control but also changes by interactive learning within the population. Inspired by the biological mechanism, integrating GA with a swarm intelligence-based method [e.g., particle swarm optimization (PSO)] might further reduce the channels for diagnosing schizophrenia by using fNIRS.

In this study, GA, PSO, and their parallel and serial hybrids were proposed with SVM for identifying schizophrenia during a VFT. This is the first study on optimizing the channels of fNIRS for diagnosing schizophrenia. The features derived by time-domain, FC, and wavelet analyses were considered. The results showed that GA and GA-dominant algorithms yielded better results, where an accuracy of 87.00% was achieved with 16 channels by using GA and wavelet feature. The use of a parallel algorithm reduced the number of channels (8 with an accuracy of 86.50% by a parallel GA–PSO optimizer on the time-domain feature). The obtained accuracy was close to that of the 52-channel system. Time-domain and wavelet features demonstrated advantages over FC feature due to schizophrenic hypofrontality, a salient characteristic in the time domain. Therefore, our findings facilitated the development of a portable fNIRS system for the diagnosis of schizophrenia during a VFT in low-resource environments.

## Materials and methods

### Participants

The dataset was provided by Peking University Sixth Hospital, Beijing, China (18). It consists of 100 SZs (male/female: 48/52, age: 30.45 ± 10.45 years) and 100 HCs (male/female: 65/35, age: 34.43 ± 12.36 years). They were all right-handed native Chinese speakers with minimum education as high school degree. Each patient was diagnosed independently by two clinical psychiatrists according to the Structured Clinical Interview for DSM-IV (19). The study was approved by the Ethics Committee of Peking University Sixth Hospital, and all participants provided written informed consent.

### Verbal fluency test

**Figure 1:**
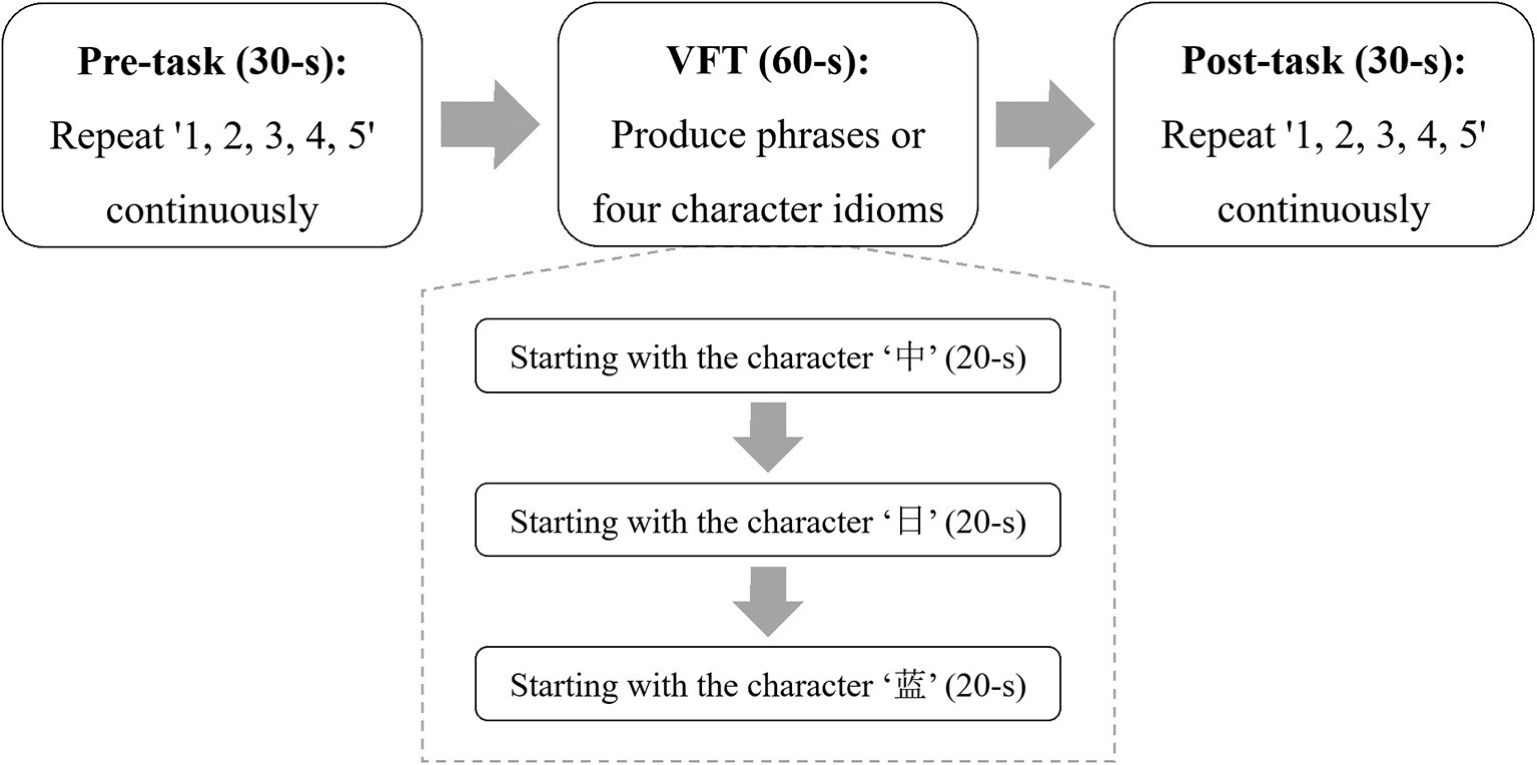
The paradigm of the VFT. During the VFT, three Chinese characters were displayed on the screen for making as many phrases as possible.

The Chinese version of the VFT (18) is illustrated in Figure 1. It included an initial 30-s pre-task baseline period, followed by a 60-s VFT period, and finally by a 30-s post-task baseline period. During the pre-task and post-task baseline periods, the participants were asked to gaze at the center of the screen, which was placed 1 m in front of them, and continuously repeat the numbers from one to five. During the VFT, three Chinese characters—“中”, “日” and “蓝”, which indicate middle, sun, and blue, respectively—were displayed on the screen successively, each for 20 s. The participants were instructed to orally coin as many phrases as possible starting with these characters, and the oxygenated hemoglobin (oxy-Hb) signal, due to its better signal-to-noise ratio (20, 21), was measured throughout the three periods.

**Figure 2:**
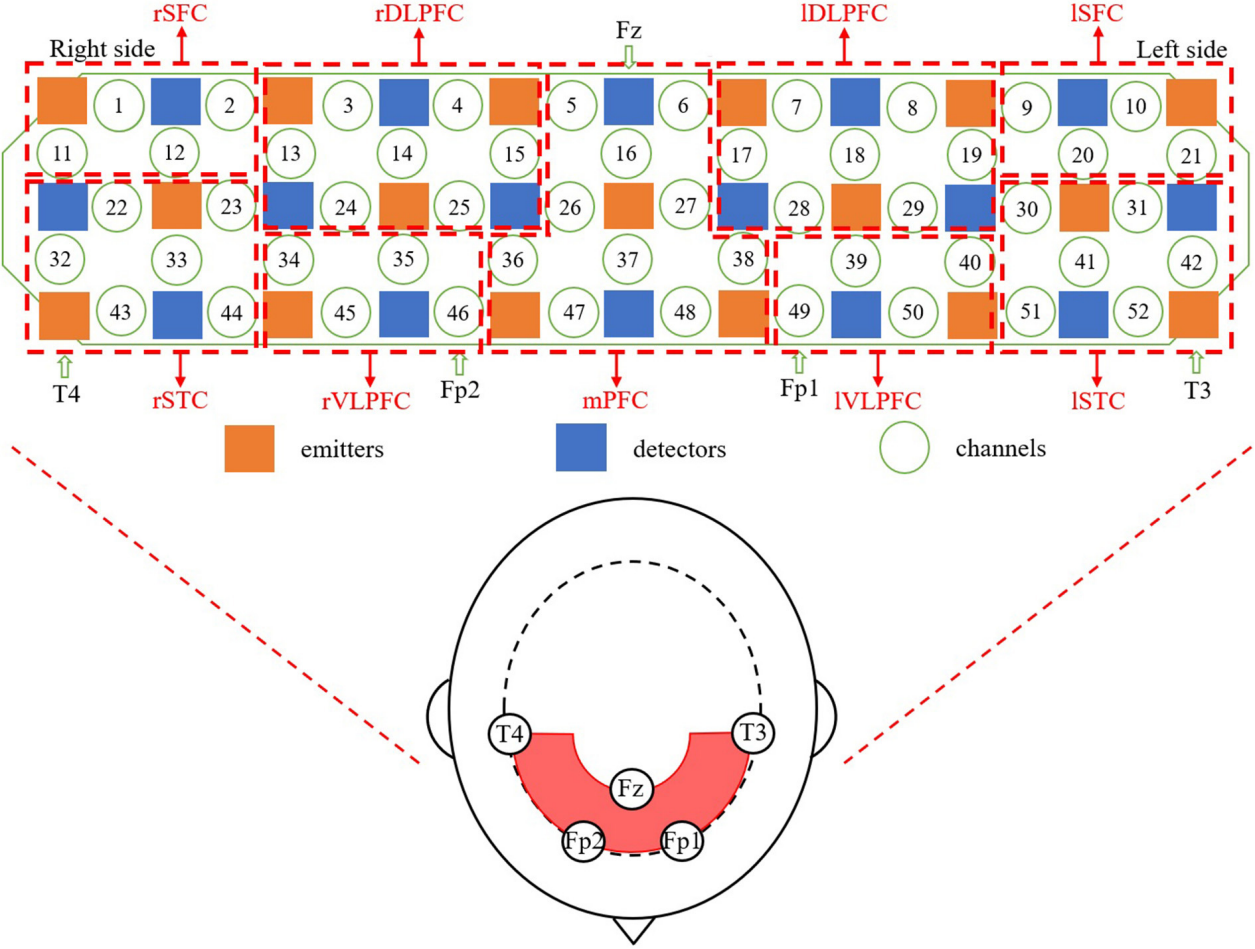
The positions of fNIRS probes and channels according to the international 10–20 system. rSFC, right superior frontal cortex; rDLPFC, right dorsolateral prefrontal cortex; lDLPFC, left dorsolateral prefrontal cortex; lSFC, left superior frontal cortex; rSTC, right superior temporal cortex; rVLPFC, right ventrolateral prefrontal cortex; mPFC, medial prefrontal cortex; lVLPFC, left ventrolateral prefrontal cortex; lSTC, left superior temporal cortex.

**Figure 3:**
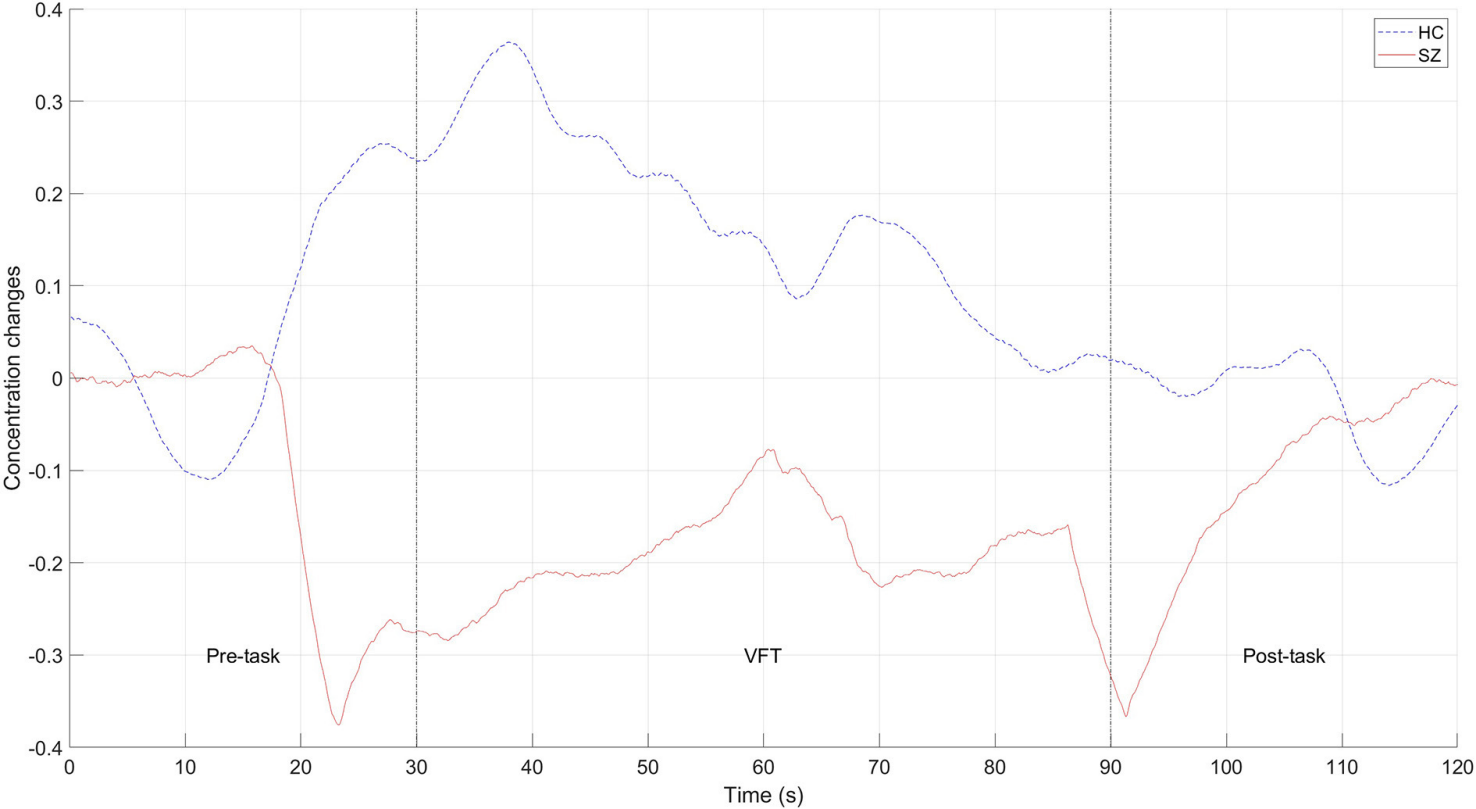
Raw signals of a random HC and a random SZ in channel 19 for example.

ETG-4000 (Hitachi Medical Co., Japan) with 52 channels was used in the experiment. As shown in Figure 2, 17 emitters and 16 detectors were positioned on the prefrontal and temporal regions based on the international 10–20 system. The sampling rate was 10 Hz. Raw signals of a random HC and a random SZ in channel 19 are shown in Figure 3 for example. The 60-s oxy-Hb signals recorded during the VFT were organized as a matrix of 200 × 52 × 600 (number of participants × number of channels × number of signal points) for further analysis.

### Signal processing pipeline

**Figure 4:**
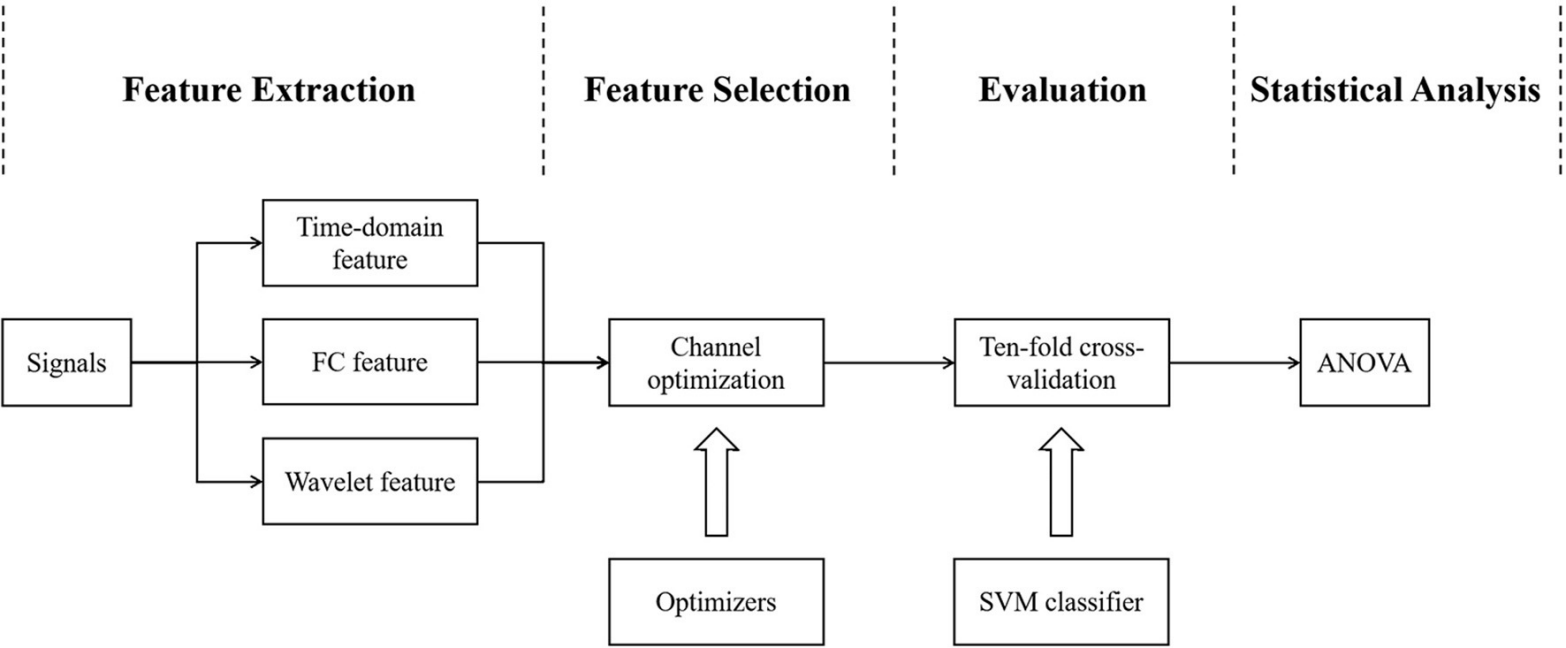
Pipeline for signal processing. GA, PSO and their parallel and serial hybrid optimizers were used for channel optimization on three fNIRS features, respectively. SVM was chosen as the classifier. The classification performance of the selected features and classifiers was evaluated by ten-fold cross-validation. Statistical analysis was performed on the accuracy and the number of channels.

A low-pass filter with a cutoff frequency of 0.6 Hz was used to remove non-physiological noises and motion artifacts (22). Consequently, temporal average, channel-wise FC of oxy-Hb within the 60-s task period were calculated. Raw signals (without prefiltering) were used for wavelet analysis. Based on the features calculated using time-domain, FC, and wavelet analysis, GA, PSO, and their parallel and serial hybrids were employed to optimize channel selection. SVM was the most suitable classifier in discriminating schizophrenia (12). The performance of the methods (including the selected features and the classifiers) was evaluated by ten-fold cross-validation. The pipeline for signal processing is shown in Figure 4.

### Feature extraction

Time-domain analysis: Mean values of the signals from each channel recorded during the VFT for each participant were computed. The results were normalized by subtracting the mean and dividing the standard deviation from all the participants.

FC analysis: Pearson's correlation coefficient was calculated to evaluate the FC between two channels.

**Figure 5:**
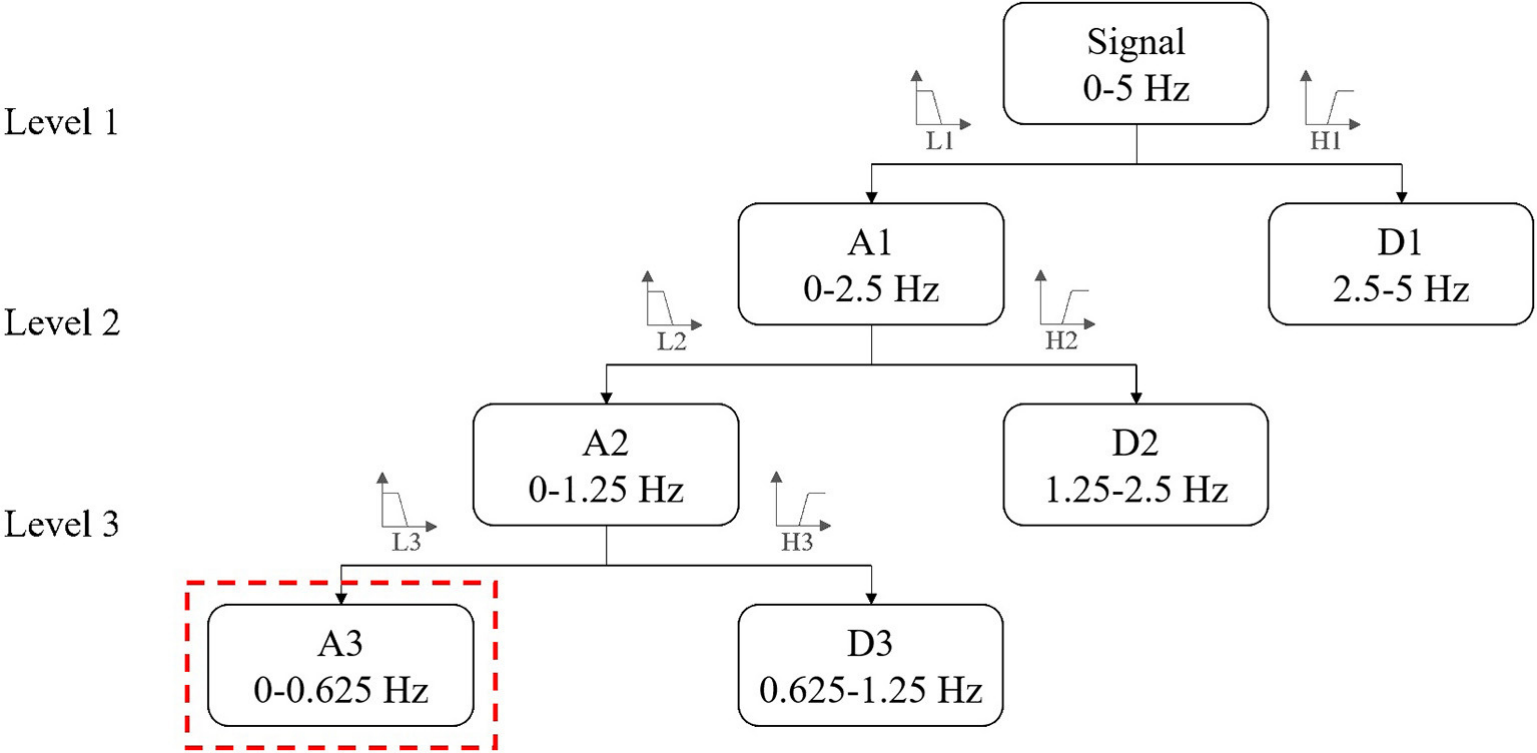
The three-level decomposition tree for discrete wavelet transform. A3 with the frequency range of 0–0.625 Hz was selected to compute wavelet energy.

Wavelet analysis: A three-level decomposition tree was used for discrete wavelet transform. Daubechies 5 was selected as the mother wavelet because of its similarity to hemodynamic response (23). As the sampling rate was 10 Hz, the frequency range for the investigation was 0–5 Hz. The procedure is illustrated in Figure 5. Time-domain and wavelet features were provided in the Supplementary Material (Supplementary Tables 1, 2, respectively).

### Encoding scheme

For feature selection by using GA and PSO, the individuals in GA and particles in PSO needed to be encoded properly. In GA, each individual was represented by a binary string of length D = 52, where “0” and “1” indicated that the corresponding channel was removed and selected, respectively. In PSO, the position of each particle was represented by a D-dimensional vector (D = 52) in which each element was distributed in [0,1] and changed into a binary format according to the threshold of 0.5, with “1” denoting the selection of the corresponding channel and “0” indicating the removal of the channel.

### Canonical GA, PSO, and their hybrids

#### GA

GA is inspired by evolution (24). In each generation, better-performing individuals are selected as parents to produce offspring by crossover (crossover rate = 0.8), thereby providing individuals with higher fitness. In addition, mutation (mutation rate = 0.01) is applied to increase the individual diversity. The fitness of every individual is evaluated by the fitness function, which is defined as


(1) $$\begin{array}{l} Fitness\;value\;=Classification\;accuracy\\ \;+0.01\times \;(Number\;of\;“0”) \end{array}$$

where the weight of the number “0” is set as 0.01 to facilitate the selection of fewer channels when two solutions have the same classification accuracy.

#### PSO

PSO simulates the social behavior of bird flocks, with the concept extended to particles flying to potential solutions through hyperspace and accelerating toward “better” solutions (25). Let *P*_*best*_ denote the previous best positions of the particles, and *G*_*best*_ denotes the global best position found by the swarm. Let *p*_*i,d*_ represent the *P*_*best*_ of particle *i* at dimension *d* (*d* = 1, 2, …, *D*) and *g*_*d*_ represent the *G*_*best*_ of particle *i* at dimension *d*. The velocity and position of *i*-th particle (*i* = 1, 2, …, *N*, where *N* is the population size) at dimension *d* are represented as *v*_*i,d*_ and *x*_*i,d*_, respectively. Then, the update for the *d*-th dimension of particle *i* can be defined as


(2) $$\begin{array}{r} v_{i,d}=\omega •v_{i,d}+c_{1}•r_{1,d}•(p_{i,d}-x_{i,d})+c_{2}•r_{2,d}•(g_{d}-x_{i,d}) \end{array}$$

(3) $$\begin{array}{r} x_{i,d}=x_{i,d}+v_{i,d} \end{array}$$

where ω = 0.9 is the inertia weight, *c*_1_ = *c*_2_ = 2, and *r*_1, *d*_ and *r*_2, *d*_ are random numbers uniformly distributed in [0,1]. The velocities of particles are limited to [−0.5, 0.5].

#### Hybrid of GA and PSO

In reality, animals' foraging behavior is controlled by interactive social learning and genetics, and can be explained by its evolutionary history (26). For example, bees' foraging paths are optimized according to sensitivity to the color and smell of flowers. A hybrid of GA and PSO, combining the biological natures of both, can facilitate the optimization process. To specify, the hybrid algorithms can take either serial or parallel forms.

##### Parallel algorithms

**Figure 6:**
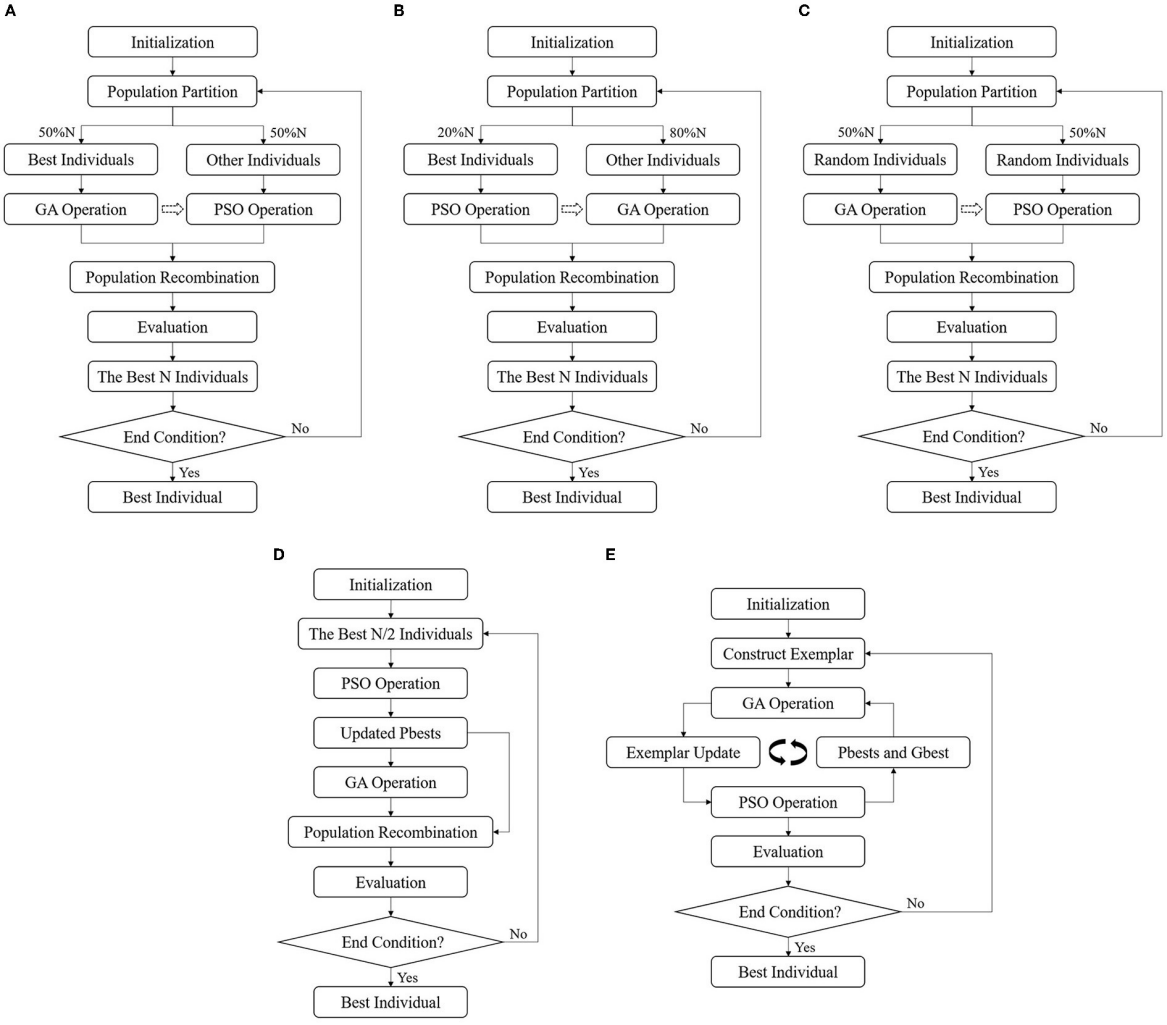
The paradigms of five hybrid algorithms. **(A)** pGAPSO-I: the best 50% individuals were assigned to GA; **(B)** pGAPSO-II: the best 20% individuals were allocated to PSO; **(C)** pGAPSO-III: the individuals were randomly distributed to GA and PSO with equal chance; **(D)** sPSOGA: PSO was first executed and then GA was applied; **(E)** sGAPSO: GA was implemented before PSO.

The parallel hybrid algorithms might differ at two stages, i.e., the population partition and information exchange between two optimizers. Therefore, three variants have been considered as pGAPSO-I/II/III. To specify, in pGAPSO-I, the best 50% individuals were assigned to GA, and the best 20% individuals were allocated to PSO in pGAPSO-II, while in pGAPSO-III the individuals were randomly distributed to GA and PSO. The specific partition of the individuals in I and II was adopted from the optimized parameters from previous studies (27, 28). The pipelines of the parallel GA-PSO algorithms are shown in Figures 6A–C.

##### Serial algorithms

The sequence of GA and PSO may vary as the population is firstly transferred to GA or PSO.

In particular, by mimicking the maturing phenomenon in nature, serial PSO-GA (sPSOGA) adopts half of the best-performing individuals for updating by PSO, whereas the other half is discarded. The updated Pbest individuals are then used for GA operation to generate better offspring. Both Pbest individuals and their offspring constitute the next population. The paradigm of sPSOGA is shown in Figure 6D.

Alternatively, inspired by the foraging behavior of birds, which is influenced by genetic control and learned from peers (29), GA was implemented before PSO (serial GA-PSO, sGAPSO): GA was first implemented using Pbest and Gbest to construct exemplars, which were then used for PSO operation to overcome premature convergence, as shown in Figure 6E.

#### Parametric setting

The maximum number of iterations was 200 for all algorithms. The population size *N* was 50 because previous studies demonstrated that population size close to the dimension of the problem (52 in this study) worked well (30). The parameters of GA and PSO were adopted from previous studies (29, 31) and used for optimizing real-life problems.

### Evaluation

The number of participants correctly classified as patients (TP) and healthy controls (TN) and then incorrectly classified as patients (FP) and healthy controls (FN) were calculated. The classification accuracy, sensitivity and specificity were defined as


(4) $$\begin{array}{r} Accuracy=\frac{TP+TN}{TP+TN+FP+FN} \end{array}$$

(5) $$\begin{array}{r} Sensitivity=\frac{TP}{TP+FN} \end{array}$$

(6) $$\begin{array}{r} Specificity=\frac{TN}{TN+FP} \end{array}$$

The accuracy/number of channels were subjected to two-way (feature × optimizer) analysis of variance (ANOVA). The variable “feature” included three levels: time-domain feature, FC feature, and wavelet feature. The variable “optimizer” consisted of seven levels: GA, PSO, pGAPSO-I/II/III, sGAPSO, and sPSOGA. Bootstrap was used for statistical inferences if normal distribution with equal variance was not achieved (32). When a statistically significant difference was detected, multiple comparisons were performed for each factor. Bonferroni correction was applied to minimize the likelihood of a type I error. SPSS 21.0 (IBM, Endicott, NY, USA) was used for statistical analysis in the study.

### Implementation

The parallel computing strategy of MATLAB was employed to reduce the calculation time. Codes of GA and PSO were adopted from the Wrapper-Feature-Selection-Toolbox (33). The in-house codes for the parallel and serial hybrid algorithms were available online (https://github.com/Xiadonn/Channel-Reduction-of-fNIRS). SVM was realized using the LIBSVM library, and the parameters of SVM were determined by performing a grid search (34). Two units of Intel Xeon CPU E5-2640 v4 @ 2.40GHz (20 cores in total) with 64-GB memory involved in the calculations.

## Results

### Performance of different features and optimizers

**Figure 7:**
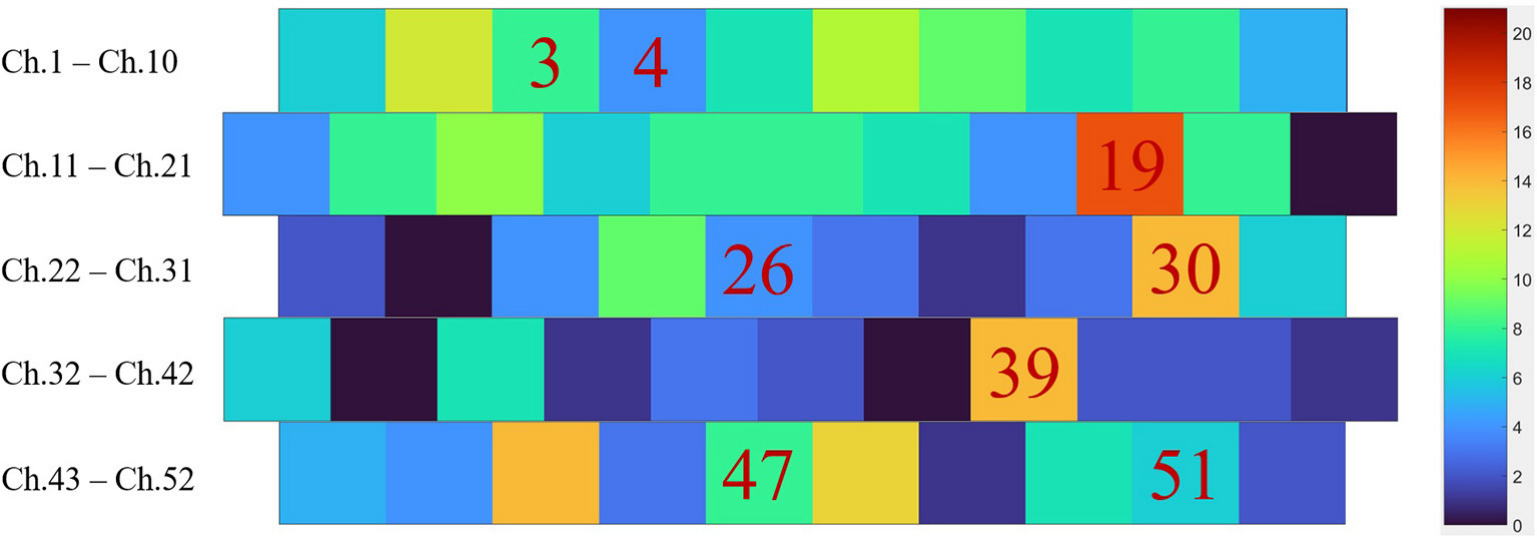
The overlay heatmap of the channels in Table 2 according to the number of their occurrences. The specific 8 channels of the optimal combination were marked.

Table 1 shows the accuracy and the number of channels obtained using different features and optimizers. The optimized channels of the best results obtained by different features and optimizers are detailed in Table 2. Figure 7 depicts the overlay heatmap of these channels according to the number of their occurrences. The best results were obtained by GA and wavelet feature, with the highest accuracy of 87.00% by using 16 channels. Furthermore, the number of channels was reduced to eight, and an accuracy of 86.50% was achieved by using pGAPSO-I on the time-domain feature. Table 3 shows the sensitivity and specificity by different features and optimizers. The comparison for sensitivity and specificity by channel optimization is in Table 4. It was found that accuracy, sensitivity and specificity were improved by channel optimization.

**Table 1:** Accuracy and the number of channels of different features and optimizers by 10-fold cross-validation, with the best results in the parathesis.

**Optimizer**	**Time-domain feature**		**FC feature**		**Wavelet feature**	
	**Accuracy**	**No. channels**	**Accuracy**	**No. channels**	**Accuracy**	**No. channels**
GA	84.75 ± 1.44% (86.50%)	16.80 ± 2.66 (13)	82.30 ± 1.32% (84.50%)	23.30 ± 2.91 (24)	84.50 ± 1.65% (87.00%)	13.80 ± 2.62 (16)
PSO	83.75 ± 1.18% (86.00%)	18.10 ± 2.77 (15)	79.40 ± 1.39% (81.50%)	23.60 ± 3.95 (17)	82.00 ± 1.11% (84.50%)	14.90 ± 1.97 (17)
pGAPSO-I	84.90 ± 1.15% (86.50%)	10.10 ± 3.67 (8)	81.25 ± 1.46% (83.50%)	17.20 ± 4.87 (16)	82.35 ± 0.75% (83.50%)	9.90 ± 1.45 (9)
pGAPSO-II	84.65 ± 0.67% (86.00%)	9.30 ± 2.11 (11)	81.45 ± 1.46% (83.50%)	13.50 ± 3.98 (11)	82.50 ± 1.37% (84.50%)	9.50 ± 1.43 (11)
pGAPSO-III	84.65 ± 1.06% (86.50%)	7.40 ± 2.27 (9)	80.75 ± 2.54% (84.00%)	19.80 ± 5.05 (15)	81.85 ± 0.71% (83.00%)	9.10 ± 2.23 (10)
sPSOGA	83.25 ± 1.59% (85.50%)	18.60 ± 4.06 (19)	80.75 ± 1.96% (85.00%)	20.40 ± 3.37 (19)	81.45 ± 1.34% (85.00%)	15.90 ± 2.85 (15)
sGAPSO	84.65 ± 1.42% (86.50%)	15.70 ± 2.54 (15)	81.80 ± 1.27% (84.00%)	24.10 ± 3.03 (21)	83.20 ± 1.53% (85.00%)	15.40 ± 2.84 (13)

**Table 2:** The specific channels corresponding to the best results obtained by different features and optimizers.

**Optimizer**	**Time-domain feature**	**FC feature**	**Wavelet feature**
GA	3 5 12 14 19 25 31 32 34 39 41 43 48	2 5 7 8 11 12 13 15 17 18 19 20 22 24 25 27 30 31 36 45 48 50 51 52	1 3 6 7 9 10 13 16 19 29 30 32 34 39 45 48
PSO	3 5 14 19 27 30 31 34 39 40 41 43 44 45 47	2 5 6 7 9 10 12 13 15 17 20 22 25 45 46 47 51	1 2 6 8 9 11 14 16 19 24 29 30 32 34 39 45 48
pGAPSO-I	3 4 19 26 30 39 47 51	2 7 9 13 14 15 16 17 20 25 26 30 36 45 51 52	3 6 7 10 19 34 39 46 48
pGAPSO-II	2 6 16 19 30 37 39 43 44 47 50	2 5 6 8 13 15 17 19 20 30 45	1 3 6 19 27 30 32 35 39 45 48
pGAPSO-III	4 13 14 19 20 31 39 47 48	2 4 7 12 14 15 16 17 25 26 31 45 48 50 51	1 2 6 15 19 30 39 45 47 48
sPSOGA	2 4 6 7 8 10 16 18 19 25 31 34 36 37 39 43 44 47 50	2 7 8 9 11 12 13 15 16 17 18 19 20 25 28 30 45 49 50	1 3 6 9 10 13 19 26 30 32 34 39 40 45 48
sGAPSO	2 5 8 12 16 19 20 24 25 39 43 44 47 48 50	2 5 7 8 9 11 12 13 15 17 18 19 20 25 30 42 45 46 48 50 51	1 3 6 9 12 13 24 29 30 32 39 45 48


**Table 3:** Sensitivity and specificity of different features and optimizers by 10-fold cross-validation.

**Optimizer**	**Time-domain feature**		**FC feature**		**Wavelet feature**	
	**Sensitivity**	**Specificity**	**Sensitivity**	**Specificity**	**Sensitivity**	**Specificity**
GA	87.40 ± 1.43%	82.10 ± 2.96%	80.80 ± 2.04%	83.80 ± 1.69%	91.70 ± 1.70%	77.30 ± 2.06%
	(91.00%)	(82.00%)	(84.00%)	(85.00%)	(94.00%)	(80.00%)
PSO	85.40 ± 2.01%	82.10 ± 2.77%	78.50 ± 2.46%	80.30 ± 3.33%	90.40 ± 1.51%	73.60 ± 2.37%
	(87.00%)	(85.00%)	(80.00%)	(83.00%)	(91.00%)	(78.00%)
pGAPSO-I	86.20 ± 1.40%	83.60 ± 2.55%	79.00 ± 2.94%	83.50 ± 2.32%	88.90 ± 2.08%	75.80 ± 1.99%
	(86.00%)	(87.00%)	(85.00%)	(82.00%)	(90.00%)	(77.00%)
pGAPSO-II	85.30 ± 1.16%	84.00 ± 1.76%	80.10 ± 3.38%	82.80 ± 3.36%	89.60 ± 2.22%	75.40 ± 3.24%
	(85.00%)	(87.00%)	(80.00%)	(87.00%)	(90.00%)	(79.00%)
pGAPSO-III	85.70 ± 0.95%	83.60 ± 2.22%	78.90 ± 3.14%	82.60 ± 3.34%	90.40 ± 1.58%	73.30 ± 2.11%
	(87.00%)	(86.00%)	(79.00%)	(89.00%)	(88.00%)	(78.00%)
sPSOGA	86.20 ± 2.25%	80.30 ± 2.91%	78.00 ± 3.56%	83.50 ± 3.78%	89.90 ± 1.52%	73.00 ± 2.16%
	(90.00%)	(81.00%)	(84.00%)	(86.00%)	(92.00%)	(78.00%)
sGAPSO	85.90 ± 2.08%	83.40 ± 2.95%	81.20 ± 2.20%	82.40 ± 0.97%	90.30 ± 1.70%	76.10 ± 2.96%
	(86.00%)	(87.00%)	(85.00%)	(83.00%)	(91.00%)	(79.00%)

*The values in parathesis corresponded to the results using optimal channel combination*.

**Table 4:** Comparison of the 10-fold cross-validation results using different features before and after channel optimization.

	**Time-domain feature**	**FC feature**	**Wavelet feature**
Accuracy	76.50/86.50% (8)	66.00/85.00% (19)	74.00/87.00% (16)
Sensitivity	83.00/86.00%	66.00/84.00%	86.00/94.00%
Specificity	70.00/87.00%	66.00/86.00%	62.00/80.00%

*The data were organized as: result with 52 channels/the best result by channel optimization, with the number of channels in the parathesis*.

### Statistical analysis of accuracy with different features and optimizers

As the interaction between the feature and optimizer was not statistically significant (*F* = 1.427, *p* = 0.156, η^2^ = 0.083), the additive model was used for statistical analysis of classification accuracy. Results showed that both feature (*F* = 92.215, *p* < 0.001, η^2^ = 0.479) and optimizer (*F* = 8.476, *p* < 0.001, η^2^ = 0.202) significantly affected the accuracy. Among three features, the time-domain feature performed better than the wavelet feature (*p* < 0.001, mean difference (MD) = 1.821%), which, in return, performed better than the FC feature (*p* < 0.001, MD = 1.450%). In the case of optimizers, GA performed better than PSO (*p* < 0.001, MD = 2.133%), pGAPSO-III (*p* = 0.003, MD = 1.433%), and sPSOGA (*p* < 0.001, MD = 2.033%), while no significant difference was noted between GA and pGAPSO-I/II (*p* = 0.134/0.174) and between GA and sGAPSO (*p* = 1.000). sGAPSO performed significantly better than PSO (*p* = 0.001, MD = 1.500%) and sPSOGA (*p* = 0.004, MD = 1.400%), and no significant difference was found between sGAPSO and pGAPSO-I/II (*p* = 1.000 for both) and between sGAPSO and pGAPSO-III (*p* = 0.656). No significant difference was observed between sPSOGA and PSO (*p* = 1.000) and between sPSOGA and pGAPSO-I/II/III (*p* = 0.134/0.102/1.000). No significant difference was noted between pGAPSO-I/II/III (*p* = 1.000 for all). However, pGAPSO-II performed significantly better than PSO (*p* = 0.044, MD = 1.150%), and no significant difference was found between PSO and pGAPSO-I/III (*p* = 0.058/1.000).

In conclusion, the time-domain feature yielded the best accuracy, followed by the features from wavelet and FC. GA, pGAPSO-II, and sGAPSO exhibited similar performances in terms of accuracy, outperforming PSO, pGAPSO-I/III, and sPSOGA.

### Statistical analysis of the number of channels between different features and optimizers

As the interaction between the feature and optimizer was significant (*F* = 3.588, *p* < 0.001, η^2^ = 0.186), the interaction model was applied for statistical analysis of the number of channels. Both feature (*F* = 121.452, *p* < 0.001, η^2^ = 0.562) and optimizer (*F* = 39.280, *p* < 0.001, η^2^ = 0.555) significantly influenced the number of channels. Among different features, FC feature utilized more channels than time-domain and wavelet features (*p* < 0.001 for both; MD = 6.56 and 7.63, respectively), and no significant difference was observed between time-domain and wavelet features (*p* = 0.134). No significant difference was found between the serial hybrid algorithm and GA and between the serial hybrid algorithm and PSO (*p* = 1.000 for both), while all of them required much more channels than pGAPSO-I/II/III (*p* < 0.001, MD ≥ 5.57). No significant difference was noted between pGAPSO-I and II (*p* = 0.946) and between pGAPSO-I/II and III (*p* = 1.000 for both).

To summarize, channel reduction by using time-domain and wavelet features was similar, and both of them were superior to the FC feature. The ability of channel reduction by pGAPSO-I/II/III was similar and outperformed the other optimizers.

## Discussion

### Accuracy achieved using different features and optimizers

**Figure 8:**
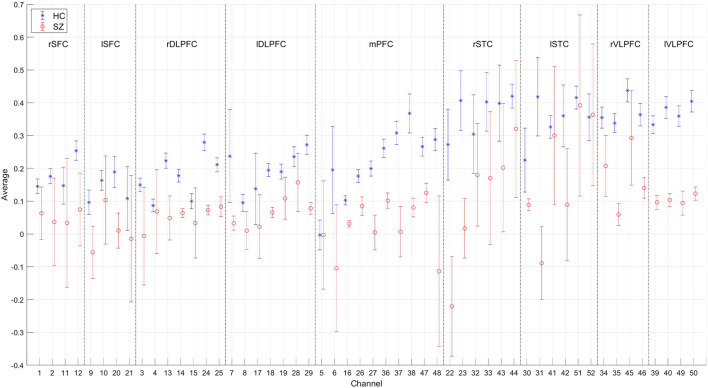
Time-domain feature of 52 channels averaged for the HCs and SZs during the VFT, respectively. The error bars were drawn with standard errors.

**Figure 9:**
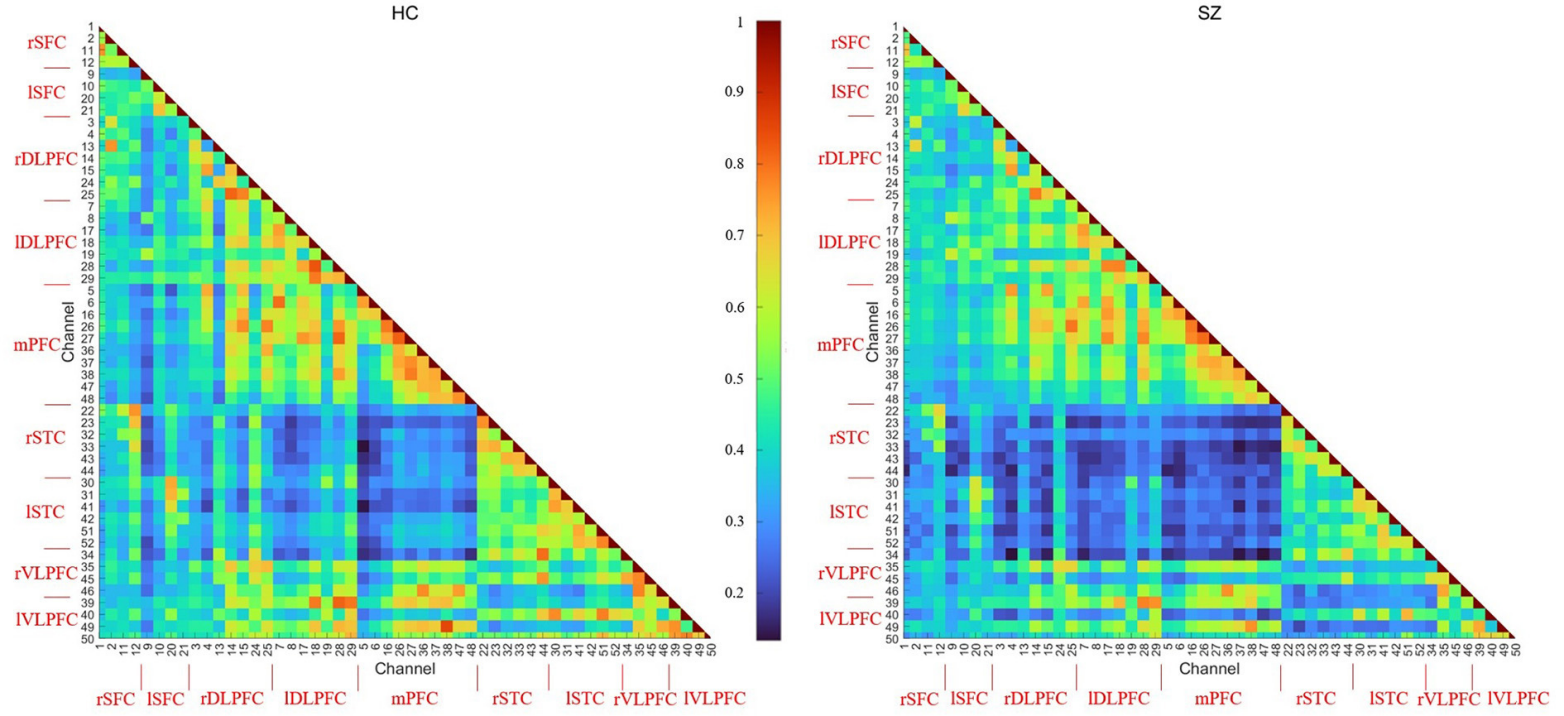
FC feature of 52 channels averaged for the HCs and SZs during the VFT, respectively.

**Figure 10:**
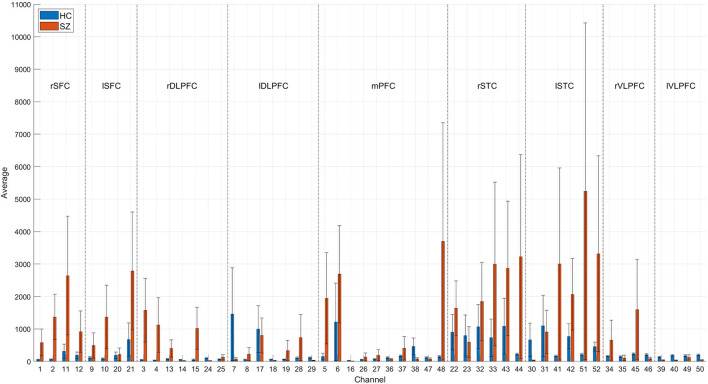
Wavelet feature of 52 channels averaged for the HCs and SZs during the VFT, respectively. The error bars were drawn with standard errors.

Temporal average, pair-wise FC, and wavelet feature are shown in Figures 8–**10**, respectively. The results were averaged for the HCs and SZs during the VFT. The reduced activation within SZs was salient in time-domain and wavelet features. The results were consistent with hypofrontality (reduced frontal cortical activation), which is frequently reported in schizophrenia (35). This has been demonstrated by many practices as the primary hemodynamic effect was widely used in identifying schizophrenia. In contrast, FC measured the regional and interregional interactions. We hypothesized that SZs were incapable of modulating the segregation and integration of hemodynamic activities from various brain regions, seemingly indiscernible compared to time-domain and wavelet features (Figure 9 vs. Figures 8, 10). Moreover, the difference between HCs and SZs in terms of wavelet features was significant, resulting in the analysis based on wavelet features yielding the highest accuracy.

Both GA and PSO are based on nature-inspired stochastic searching techniques. GA is popular for its superior global searching ability and PSO for its local exploration ability. However, GA and PSO have certain limitations, such as a lack of diversity resulting in a suboptimal solution or a slow convergence rate (30). In this study, GA and GA-dominant (pGAPSO-II and sGAPSO) optimizers exhibited superior performances than PSO-dominant optimizers in terms of accuracy, indicating that the search volume might contain many local optima, thus being liable to trap the PSO particles during local exploration. A previous study substantiated this assumption: the discriminating ability achieved by signals from an individual channel was rather similar; for example, the highest accuracy of the first five channels ranged from 77.50 to 82.50% (12). Thus, different combinations can achieve similar performances. Serial algorithms performed worse compared with parallel algorithms. This can be attributed to the PSO process leading to premature convergence of the individuals, which can be avoided in parallel algorithms, or the diversity produced by GA getting obscured by the follow-up PSO step (36).

Parallel algorithms achieved higher accuracy compared to GA while using fewer channels, thereby showing the importance of incorporating PSO in the optimizer because it facilitates local exploration in a feature hyperspace comprising very subtle differences.

The analysis provided insights on future work of discriminating schizophrenia with fewer fNIRS channels during a VFT by using time-domain/wavelet features and evolutional algorithm-dominated parallel algorithms while achieving an overall accuracy comparable to that of contemporary 52-channel fNIRS [70–90% (12–14, 37–40)]. Furthermore, Table 5 lists the detailed comparison between this work and similar studies aiming at channel reduction. We achieved an accuracy of 86.50% with 8 channels by using pGAPSO-I, SVM, and time-domain average features derived from oxy-Hb signals during the VFT, outperforming other studies. This result demonstrates the effectiveness of discriminating SZs and HCs with fewer channels, promoting the application of portable fNIRS devices in clinical scenarios. By then, for people with developmental dyslexia/specific language impairment the Structured Clinical Interview for DSM-IV will be used for co-diagnosis.

**Table 5:** Comparison between this study and similar 52-channel fNIRS studies aiming at channel reduction in schizophrenic identification.

**References**	**Task**	**Signal**	**Feature**	**Feature selection method**	**Classifier**	**Accuracy**	**Number of channels**	**Specific channels**
Chuang et al. (39)	VFT	oxy-Hb	Time-domain average	Two-sample Kolmogorov–Smirnov test	K-means clustering	71.72%	6	23 29 31 40 42 52
Ji et al. (13)	VFT	oxy-Hb	FC	Seed-based FC analysis	SVM	89.67%	26	3–4, 15–18, 24–29, 34–38, 43–50 and 52
Chen et al. (17)	One-back memory task	total-Hb	Activation degree in time domain	Independent sample *t*-test	SVM	89.50%	39	1–17, 19–21, 26, 30–31, 33, 35, 37, 39–50 and 52
Ours	VFT	oxy-Hb	Wavelet energy	GA	SVM	87.00%	16	1 3 6 7 9 10 13 16 19 29 30 32 34 39 45 48
			Time-domain average	pGAPSO-I	SVM	86.50%	8	3 4 19 26 30 39 47 51

*total-Hb, relative concentration of total hemoglobin*.

Tables 3, 4 indicates enhanced classification performance by optimization. It could be attributed to the elimination of the irrelevant features (e.g., noise, outliers, redundant features), which affected the system performance (41).

### Optimized channel combinations

The best channel sets (Table 2) varied for different features and optimizers, but some common points existed. First, channels in lDLPFC and mPFC were manifested in every combination; in particular, most cases contained no less than two channels in mPFC, with one exception (sPSOGA on FC feature). Second, lDLPFC acted as key nodes in FC analysis because more than two channels in lDLPFC were included in the results of the analysis. Third, lDLPFC, mPFC, and rDLPFC appeared in most of the cases (with the exception of pGAPSO-II in the time-domain feature). Fourth, channels in ISTC were found in most of the cases (with exceptions of sGAPSO in the time-domain feature and pGAPSO-I in the wavelet feature), while channels in lVLPFC were missing in FC analysis (PSO and pGAPSO-I/II). The results were consistent with the neurophysiological function of the individual cortex; for example, mPFC plays an important role in decision making and short- and long-term memory (42). It coordinated bilateral DLPFC and VLPFC functions (43), which were recruited in cognitive control and self-control (44–46), response inhibition, and goal-appropriate response selection (47). Further, dysfunction in lDLPFC is related to the severity of schizophrenic symptoms and conceptual disorganization, which are not related to antipsychotic treatment (48). Therefore, it was reasonable to observe abnormalities in these cortices and the importance of lDLPFC as a key node for FC feature. In addition, because lSTC was critical for language ability (49), the dysfunction of this region could be manifested in the VFT. Remarkedly, its absence was observed only in two cases with mediocre accuracy (sGAPSO on the time-domain feature and pGAPSO-I on the wavelet feature). The abovementioned findings correlated with the cortical abnormalities of schizophrenia observed across different imaging modalities.

### Limitations

This study has some limitations. Firstly, the staging of schizophrenia was not conducted due to the lack of labels on the stages of schizophrenia. Secondly, most of the parameters were adopted from previous studies and were not further fine-tuned. Further extensive tuning of the parameters can be conducted in future work.

## Conclusion

In this paper, two nature-inspired optimizers, GA and PSO, as well as their parallel and serial hybrid combinations, were used to simplify the number of fNIRS channels employed for discriminating schizophrenia during a VFT. The optimization was conducted on time-domain, FC, and wavelet features of 52-channel fNIRS signals. By using the time-domain feature, pGAPSO-I, and SVM, we achieved an accuracy of 86.50% (ten-fold cross-validation) with 8 channels. Based on the results, the impact of specific features and optimizers on the classification results was discussed. Furthermore, the results provided insights into identifying patients with schizophrenia by using fewer channels, thus promoting the development of portable fNIRS diagnostic systems in low-resource environments.

## Data availability statement

The original contributions presented in the study are included in the article/supplementary materials, further inquiries can be directed to the corresponding author/s.

## Ethics statement

The studies involving human participants were reviewed and approved by the Ethics Committee of Peking University Sixth Hospital. The patients/participants provided their written informed consent to participate in this study.

## Author contributions

TW and DX initiated and designed the study. WQ was responsible for data collection. DX implemented the codes, performed statistical analyses, and drafted the manuscript. TW revised the final version. All authors have approved the final version.

## Funding

This study was supported in part by grants from the National Natural Science Foundation Project (No. 61971445) and the National Key Research and Development Program of China (Nos. 2019YFF0216302 and 2018YFC1314200).

## Conflict of interest

The authors declare that the research was conducted in the absence of any commercial or financial relationships that could be construed as a potential conflict of interest.

## Publisher's note

All claims expressed in this article are solely those of the authors and do not necessarily represent those of their affiliated organizations, or those of the publisher, the editors and the reviewers. Any product that may be evaluated in this article, or claim that may be made by its manufacturer, is not guaranteed or endorsed by the publisher.
